# Regulatory Landscape of the *Pseudomonas aeruginosa* Phosphoethanolamine Transferase Gene *eptA* in the Context of Colistin Resistance

**DOI:** 10.3390/antibiotics12020200

**Published:** 2023-01-18

**Authors:** Matteo Cervoni, Davide Sposato, Alessandra Lo Sciuto, Francesco Imperi

**Affiliations:** 1 Department of Science, Roma Tre University, 00146 Rome, Italy; 2 IRCCS Fondazione Santa Lucia, 00179 Rome, Italy; 3 NBFC, National Biodiversity Future Center, 90133 Palermo, Italy

**Keywords:** *arn* genes, EptA, L-aminoarabinose, lipid A, phosphoethanolamine transferase, polymyxin, reporter strain, resistance, spontaneous mutations, transposon mutagenesis

## Abstract

*Pseudomonas aeruginosa* has the genetic potential to acquire colistin resistance through the modification of lipopolysaccharide by the addition of 4-amino-4-deoxy-L-arabinose (L-Ara4N) or phosphoethanolamine (PEtN), mediated by the *arn* operon or the *eptA* gene, respectively. However, in vitro evolution experiments and genetic analysis of clinical isolates indicate that lipopolysaccharide modification with L-Ara4N is invariably preferred over PEtN addition as the colistin resistance mechanism in this bacterium. Since little is known about *eptA* regulation in *P. aeruginosa*, we generated luminescent derivatives of the reference strain *P. aeruginosa* PAO1 to monitor *arn* and *eptA* promoter activity. We performed transposon mutagenesis assays to compare the likelihood of acquiring mutations leading to *arn* or *eptA* induction and to identify *eptA* regulators. The analysis revealed that *eptA* was slightly induced under certain stress conditions, such as arginine or biotin depletion and accumulation of the signal molecule diadenosine tetraphosphate, but the induction did not confer colistin resistance. Moreover, we demonstrated that spontaneous mutations leading to colistin resistance invariably triggered *arn* rather than *eptA* expression, and that *eptA* was not induced in resistant mutants upon colistin exposure. Overall, these results suggest that the contribution of *eptA* to colistin resistance in *P. aeruginosa* may be limited by regulatory restraints.

## 1. Introduction

The increase in antibiotic resistance among bacterial pathogens is becoming a critical global crisis, mainly due to the emergence of multidrug-resistant (MDR) strains, especially among Gram-negative bacterial pathogens [1]. One of the few remaining options for treating life-threatening infections caused by MDR Gram-negative bacteria is colistin, a cationic antibiotic belonging to the polymyxin family that is now considered a “last-resort” antibacterial drug [2,3]. Unfortunately, the use of colistin in human and veterinary medicine has led to the emergence and spread of colistin-resistant isolates, often leaving clinicians without suitable resources to treat patients [4,5].

Colistin is a cyclic heptapeptide with a tripeptide side chain acylated at the N terminus, whose positive charge is due to the presence of multiple α,γ-diaminobutyric acid residues [6]. According to the “self-promoted uptake pathway” model, the initial binding of colistin with the outer membrane (OM) of Gram-negative bacteria occurs via electrostatic interactions with the negatively charged phosphate groups of the lipid A moiety of lipopolysaccharide (LPS). Then, colistin competitively displaces the divalent cations Mg^2+^ and Ca^2+^ that stabilize the LPS layer, thus weakening the OM and facilitating its uptake [5,7]. Recently, it was demonstrated that colistin can also interact with LPS molecules that are transiently present in the cytoplasmic membrane before transport to the OM, accounting for membrane destabilization, loss of cytoplasmatic content, and finally cell lysis [8].

In many Gram-negative bacteria, polymyxin resistance relies on the remodelling of lipid A by the covalent addition of positively charged molecules, such as phosphoethanolamine (PEtN) and 4-amino-4-deoxy-L-arabinose (L-Ara4N), which decrease the negative charge of lipid A and reduce the affinity of polymyxins for LPS [9]. Other polymyxin-resistance mechanisms have been proposed, such as lipid A glycosylation or acylation, capsule overproduction, expression of efflux pumps, and overexpression of basic outer membrane proteins that can bind and mask the divalent cation-binding sites of LPS [9,10]. However, their contribution to polymyxin resistance remains unclear.

*Pseudomonas aeruginosa* is an opportunistic pathogen that causes both acute and chronic infections with limited therapeutic options [11]. In this bacterium, colistin resistance is always associated with the induction of the *arn* operon, which is responsible for L-Ara4N-dependent modification of lipid A and is controlled by a complex regulatory network involving several two-component systems (TCSs) [4,12,13]. Indeed, mutations in TCSs that control the expression of *arn* genes have frequently been identified in colistin-resistant strains [12,13,14,15,16]. By combining reverse genetics with experimental evolution assays, it has been confirmed that a functional lipid A aminoarabinosylation pathway is essential for the acquisition of colistin resistance in vitro, in clinical *P. aeruginosa* isolates and reference samples [15,16]. On the other hand, analysis of recombinant *P. aeruginosa* strains constitutively expressing *arn* genes revealed that lipid A aminoarabinosylation is sufficient to confer colistin resistance in some but not all *P. aeruginosa* strains [17]. This is in line with the observation that the evolution towards high levels of colistin resistance also involves mutations in other genetic loci that are not directly related to lipid A modification, and are probably required to improve and/or support the effect of L-Ara4N-modified lipid A on colistin resistance [15]. Nonetheless, these studies overall confirm lipid A aminoarabinosylation as the main colistin-resistance mechanism in *P. aeruginosa*. Accordingly, inhibitors of the Ara-4N transferase ArnT, which catalyses the last step of lipid A aminoarabinosylation, were found to restore susceptibility in colistin-resistant *P. aeruginosa* strains [18,19].

Conversely, the role of lipid A phosphoethanolamination in *P. aeruginosa* is less clear. *P. aeruginosa* has an endogenous gene, named *eptA*, which encodes a functional PEtN transferase capable of adding PEtN to lipid A [20,21]. It was reported that *eptA* gene expression in *P. aeruginosa* is induced by extracellular zinc, but not other metals, through the TCS ColRS, and that zinc-induced PEtN addition to lipid A does not confer polymyxin resistance [20]. Furthermore, Liu and colleagues observed that the introduction into *P. aeruginosa* of plasmids carrying the mobile gene *mcr-1*, which encodes the PEtN transferase MCR-1, led to colistin resistance, although the increase in colistin MIC varied between isolates [22,23]. More recently, by cloning the *eptA* and *mcr-1* genes in a plasmid for IPTG-inducible expression, it was demonstrated that (i) exogenous and endogenous PEtN transferases (MCR-1 and EptA) in *P. aeruginosa* have comparable lipid A PEtN transferase activity, (ii) both are effective in promoting colistin resistance, and (iii) the modification of lipid A with PEtN confers levels of colistin resistance comparable to those conferred by L-Ara4N-modified lipid A [21].

Given the efficacy of PEtN transferases in conferring colistin resistance in *P. aeruginosa* when ectopically expressed from plasmids [21,22,23], it is unclear why lipid A aminoarabinosylation is always preferred over lipid A phosphoethanolamination as a colistin-resistance mechanism [12,13,14,15,16]. Since little is known about the regulatory landscape of *P. aeruginosa eptA*, in this study we generated reporter strains and performed random mutagenesis screenings to evaluate the propensity of *P. aeruginosa* to acquire mutations that induce *eptA*, and to identify genes that influence *eptA* gene expression and/or colistin resistance.

## 2. Results

### 2.1. PEtN Transferases Support the Evolution of P. aeruginosa towards High Levels of Colistin Resistance

**Figure 1:**
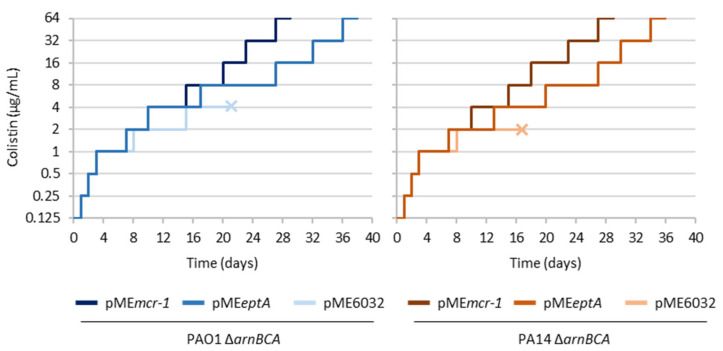
In vitro evolution assays for L-Ara4N deficient mutants of *P. aeruginosa* PAO1 and PA14 (Δ*arnBCA*) carrying plasmids for the ectopic expression of EptA (pME*eptA*) or MCR-1 (pME*mcr-1*), or the empty plasmid pME6032 as the control. Strains were evolved through serial passages in the presence of increasing concentrations of colistin (up to 64 μg/mL). Five biological replicates (independent cultures) were analyzed for each strain. The graphs show a representative curve for each strain, while the curves of the other replicates are provided in Figure S1. The “×” symbol highlights cultures that went extinct during the evolution experiment.

Previous studies demonstrated that ectopic expression of endogenous or exogenous PEtN transferases (EptA and MCR-1, respectively) is able to confer colistin resistance in *P. aeruginosa* [21,22,23]. However, evolution experiments and genomic analyses revealed that the acquisition of high levels of colistin resistance in *P. aeruginosa* requires a functional L-Ara4N lipid A modification pathway and mutations in several independent loci that synergistically produce the resistance phenotype [15,16]. Thus, to further verify the potential of PEtN to contribute to colistin resistance acquisition in *P. aeruginosa*, we performed in vitro evolution assays for L-Ara4N-defective mutants (*ΔarnBCA*) of two different reference strains (PAO1 and PA14) transformed with plasmids for expression of either EptA or MCR-1, using an empty plasmid as the control. With this aim, five independent cultures for each strain were subsequently refreshed in the presence of increasing concentrations of colistin (up to 64 µg/mL), and cultures were considered extinct when they showed no visible growth after five days. In line with previous evidence [16], the *ΔarnBCA* mutants containing the empty plasmid were unable to evolve the ability to grow at colistin concentrations higher than 2 or 4 µg/mL (Figure 1 and Supplementary Materials Figure S1), confirming the necessity of L-Ara4N-modified lipid A for the acquisition of high levels of colistin-resistance in wild-type backgrounds. In contrast, strains ectopically expressing PEtN transferases successfully evolved high levels of colistin resistance, although the evolutionary process appeared to be slightly faster in MCR-1-expressing cells compared with EptA-expressing cells (Figure 1 and Supplementary Materials Figure S1). This could be explained by the previously observed detrimental effect of ectopic EptA expression on *P. aeruginosa* growth [21]. Nevertheless, the experimental results demonstrate that PEtN transferases, including the endogenous enzyme EptA, when ectopically expressed from plasmids can support the evolution of *P. aeruginosa* towards high-level colistin resistance. This led us to hypothesize that in *P. aeruginosa* the role of the chromosomal *eptA* gene in the acquisition of colistin resistance could be limited by regulatory restraints.

### 2.2. Generation and Validation of Reporter Strains for PeptA and Parn Activity

**Figure 2:**
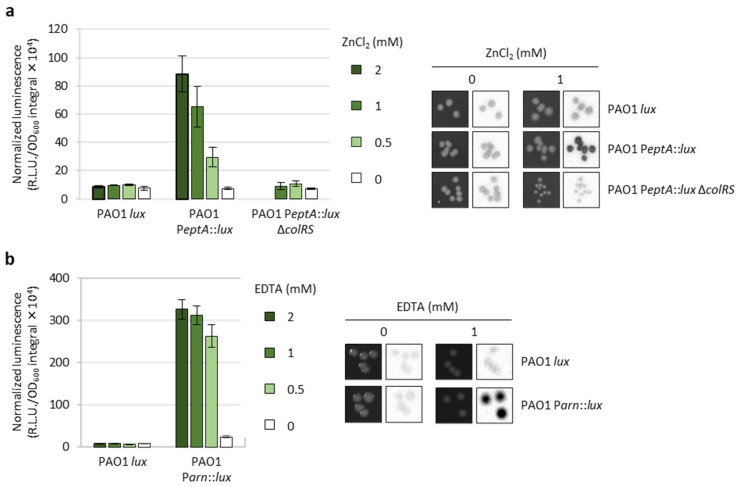
Validation of the PAO1 P*eptA::lux* and PAO1 P*arn::lux* reporter strains. (**a**) Area under the normalized luminescence curve for PAO1 *lux*, PAO1 P*eptA::lux*, and PAO1 P*eptA::lux* Δ*colRS* cultured in lysogeny broth (LB) supplemented with different ZnCl_2_ concentrations, and colony growth (left images) and luminescence (right images) of the same strains on LB agar plates in the presence or absence of 1 mM ZnCl_2_. PAO1 P*eptA::lux* Δ*colRS* did not grow at 2 mM ZnCl_2_ (data not shown and [20]). (**b**) Area under the normalized luminescence curve for PAO1 *lux* and PAO1 P*arn::lux* cultured in LB supplemented with different EDTA concentrations, and colony growth (left images) and luminescence (right images) of the same strains on LB agar plates in the presence or absence of 1 mM EDTA. Values are the mean (±SD) of three independent assays. Images are representative of three independent experiments providing similar results.

To investigate the regulatory network(s) involved in the control of *eptA* gene expression, we generated a luminescent reporter in the reference strain PAO1 (PAO1 P*eptA::lux*) harboring the *lux*CDABE operon under the control of the *eptA* promoter inserted into a neutral site of the chromosome. Similarly, we generated another reporter strain to monitor the activity of the arn promoter (PAO1 P*arn::lux*), and a control strain carrying only the *lux* operon inserted into the same chromosomal site (PAO1 *lux*). The PAO1 P*eptA::lux* reporter was validated by assessing luminescence emission in response to different concentrations of extracellular zinc (ZnCl_2_) during both planktonic and colony growth. As expected, luminescence was induced by zinc in a dose-dependent manner, and such induction was not observed upon deletion of the colRS genes (PAO1 P*eptA::lux ΔcolRS*) (Figure 2a), confirming that *eptA* regulation by zinc occurs through the TCS ColRS [20]. Interestingly, we also confirmed that zinc-mediated induction of *eptA* gene expression does not increase colistin resistance in *P. aeruginosa* [20], and observed that extracellular zinc had a negative impact on bacterial growth at *eptA*-inducing concentrations (Figure S2).

The PAO1 P*arn::lux* strain was validated by demonstrating that the luminescence emitted by this reporter was proportional to the concentration of the divalent cation chelator EDTA in the growth medium (Figure 2b), which removes Ca^2+^ and Mg^2+^ that stabilize the OM and induces the *arn* operon [24]. As expected, neither zinc nor EDTA had any effect on luminescence emission in the control strain PAO1 *lux* (Figure 2).

### 2.3. Identification of Genes Influencing eptA Promoter Activity and Evaluation of Their Impact on Colistin Resistance

To identify novel regulators of eptA gene expression, we carried out transposon-mediated random mutagenesis in PAO1 P*eptA::lux*. For comparison, transposon mutagenesis was also performed in PAO1 P*arn::lux*. Transconjugants were picked from conjugation plates, spotted onto new agar plates, and screened for luminescence emission using PAO1 P*eptA::lux* (or PAO1 P*arn::lux*), PAO1 *lux*, and the PAO1 wild type as controls representing non-induced or basal luminescence levels. Luminescence was quantified for each clone (see Section 4) and compared among clones in order to identify possible outliers producing higher or lower levels of luminescence. An example of a screening plate is provided in Figure S3.

**Figure 3:**
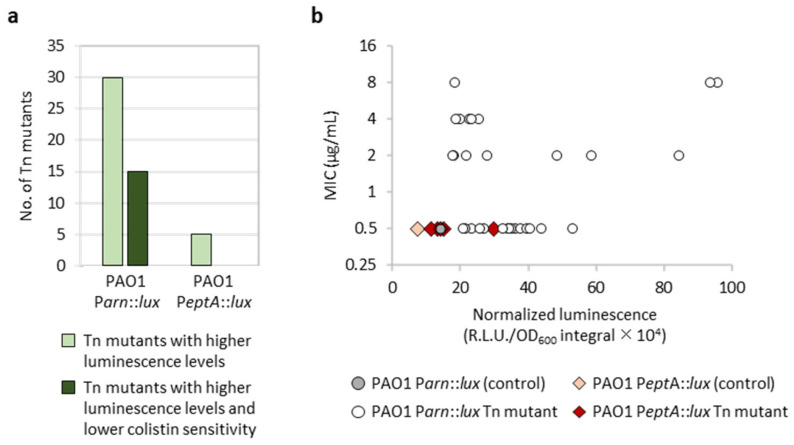
Overview of the results of the transposon mutagenesis screening. (**a**) Number of transposon mutants of PAO1 P*arn::lux* or PAO1 P*eptA::lux* showing higher luminescence emission, both on agar plates and during planktonic growth, and/or higher colistin MIC compared with the corresponding parental strain. (**b**) Correlation between P*eptA* or P*arn* activity (x axis) and colistin MIC (y axis) for the selected transposon mutants of PAO1 P*arn::lux* (circles) or PAO1 P*eptA::lux* (diamonds) and the parental strains for comparison. Luminescence levels were quantified from liquid cultures and correspond to the mean of three independent experiments.

The screening of 12,000 transposon mutants for each reporter led to identification of only clones with increased luminescence levels, probably because the *eptA* and arn promoters were poorly active under the culture conditions used for the screening (LB agar plates without any supplement) (Figure 2). The outliers identified in the screening plates were verified for luminescence emission in liquid cultures and tested by MIC assays for colistin sensitivity. Overall, we identified 30 transposon mutants for PAO1 P*arn::lux* and five for PAO1 P*eptA::lux* that emitted significantly higher levels of luminescence with respect to the corresponding controls or the other mutant clones (Figure 3). Half of the selected PAO1 P*arn::lux* transposon mutants also showed increased colistin resistance (≥4-fold increase in MIC), while colistin MIC was identical to the parental strain for all PAO1 P*eptA::lux* transposon mutants (Figure 3). Notably, we did not observe a clear correlation between Parn activity and colistin MIC (Figure 3b), suggesting that arn gene expression is not always sufficient to confer colistin resistance. This is in line with previous results obtained with evolution experiments or genetically engineered strains [15,16].

Overall, these results suggest that transposon insertions leading to increased promoter activity are more likely to occur for Parn than PeptA, leading to the proposal that the arn operon might be more connected to the *P. aeruginosa* regulatory network with respect to the *eptA* gene. Moreover, even if the number of mutants (only five) with enhanced P*eptA* activity identified in our screening is poorly indicative, the observation that none of these mutants showed any increase in colistin resistance is consistent with previous evidence that *eptA* expression and lipid A phosphoethanolamination induced by extracellular zinc are not sufficient to confer colistin resistance in *P. aeruginosa* (Figure 2a and Figure S2) [20].

Since our study was aimed at identifying novel regulators of eptA gene expression, we identified the transposon insertion site for the five P*eptA::lux* mutants showing increased luminescence emission. Transposon insertions were found in anabolic genes involved in biotin (*bioA* and *bioB*) or arginine biosynthesis (*argF*), and in the *apaH* and *rmcA* genes involved in the degradation of the intracellular signaling molecules diadenosine tetraphosphate (Ap4A) and cyclic diguanylate (c-di-GMP), respectively (Table 1) [25,26]. While the function of c-di-GMP in promoting cell aggregation and the biofilm lifestyle has been well documented in *P. aeruginosa* [27,28], less is known about the role of Ap4A in bacteria. Ap4A production is generally increased under stress, but it is still debated whether it represents a bona fide secondary messenger or a damage metabolite [29,30]. Notably, none of these genes have previously been linked to regulation of lipid A modification genes and/or colistin resistance.

### 2.4. Further Characterization of Genes Influencing eptA Promoter Activity

**Figure 4:**
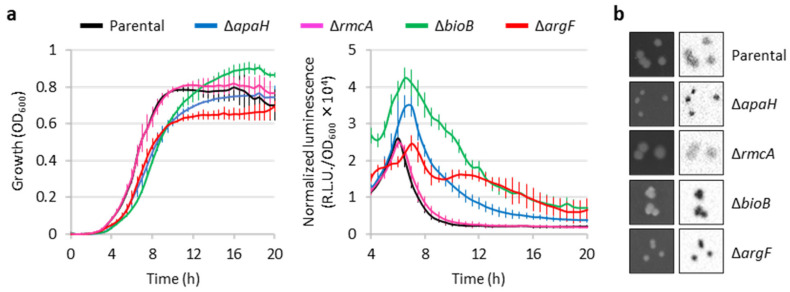
Validation of the putative *eptA* regulators by deletion mutagenesis. (**a**) Growth (left panel) and normalized luminescence curves (right panel) of PAO1 P*eptA::lux* and its deleted *apaH*, *rmcA*, *bioB*, or *argF* derivatives, cultured in LB. Values are the mean (±SD) of three independent assays. (**b**) Colony growth (left images) and luminescence (right images) of the above-mentioned strains on LB agar plates. Images are representative of three independent experiments giving similar results.

Given that transposon insertions within coding sequences are expected to cause loss of gene function, to confirm the transposon mutagenesis results we generated clean deletion mutants of the PAO1 P*eptA::lux* reporter in all the identified genes except *bioA* (Table S1), as the impact of the loss of biotin biosynthesis could be inferred from the Δ*bioB* mutant. The deletion mutants in *apaH*, *bioB*, and *argF* showed increased luminescence emission compared with the parental strain, in line with what observed for the corresponding transposon mutants (Figure 4). In contrast, the Δ*rmcA* mutant had luminescence levels comparable to the parental reporter strain, during both planktonic and colony growth. This mutant was therefore not included in the following analyses. Interestingly, the Δ*apaH*, Δ*bioB*, and Δ*argF* mutants also showed impaired and/or delayed growth (Figure 4a), indicating that the absence of biotin or arginine biosynthesis has a negative impact on growth even in a rich medium such as LB. The growth defects of the Δ*apaH* mutant could be explained by the intracellular accumulation of Ap4A, probably caused by the lack of Ap4A tetraphosphatase activity, and indeed it has been reported that high levels of Ap4A can have deleterious effects on bacterial fitness [29]. Growth and luminescence emission in deletion mutants were restored to parental levels by complementation with plasmids expressing the cognate gene (Figure S4), confirming that the observed phenotypes were caused by the lack of the genes under investigation.

**Figure 5:**
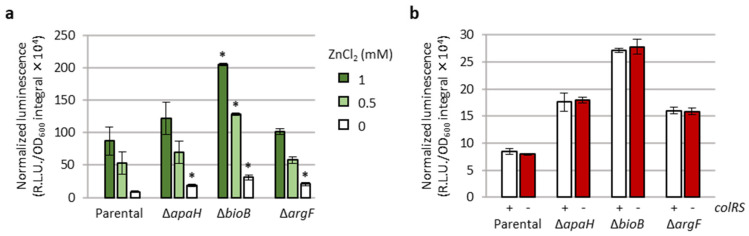
ApaH, BioB, and ArgF affect *eptA* promoter activity in a ColRS-independent manner. (**a**) P*eptA* promoter activity (area under the normalized luminescence curves) in PAO1 P*eptA::lux* and its deleted *apaH*, *bioB*, or *argF* derivatives, cultured in LB supplemented with different concentrations of ZnCl_2_. (**b**) P*eptA* promoter activity (area under the normalized luminescence curves) in the above-mentioned strains and in isogenic deletion mutants lacking the *colRS* genes, cultured in LB. Values are the mean (±SD) of three independent assays. Asterisks (*) indicate a statistically significant increase in promoter activity with respect to the parental strain cultured in the presence of the same ZnCl_2_ concentration, according to the unpaired *t* test (*p* < 0.01).

Based on the function of ApaH, BioB, and ArgF (Table 1), it is plausible to predict that these proteins have an indirect effect on *eptA* promoter activity. Since the only regulatory system demonstrated to control *eptA* transcription is the TCS ColRS, which is activated by extracellular zinc [20], we verified whether the lack of the *apaH*, *bioB*, or *argF* genes could influence zinc-mediated induction of *eptA* expression and/or whether the deletion of *colRS* genes could reduce the effect of Δ*apaH*, Δ*bioB*, or Δ*argF* mutations on *eptA* promoter activity. As shown in Figure 5a, the PAO1 P*eptA::lux* reporter was induced by extracellular zinc in a dose-dependent manner even in the absence of *apaH*, *bioB*, or *argF*. Moreover, the levels of luminescence emitted by PAO1 P*eptA::lux* Δ*apaH*, Δ*bioB*, and Δ*argF* mutants, respectively, were basically identical in the presence or absence of a functional ColRS system (Figure 5b). Overall, these results clearly indicate that ApaH, BioB, and ArgF affect P*eptA* activity in a ColRS-independent manner.

In the attempt to further characterize the impact of impaired anabolic metabolism on *eptA* gene expression, the Δ*bioB* and Δ*argF* mutants were also assayed in minimal medium. As expected, both mutants did not grow unless biotin or arginine was added to the medium, and their growth was promoted by exogenously supplied biotin or arginine in a dose-dependent manner (Figure S5), confirming the auxotrophy of the Δ*bioB* and Δ*argF* mutants for biotin and arginine, respectively. Interestingly, P*eptA* activity in these strains was found to be inversely proportional to growth rates and yields, with maximum P*eptA* induction observed at biotin or arginine concentrations that sustained growth at levels far below those of the parental prototrophic strain (Figure S5). These data indicate that depletion of arginine or biotin, rather than the lack of the specific ArgF or BioB enzymes, is the signal that triggers *eptA* gene expression. However, the mechanism of induction was not further investigated in this study.

### 2.5. The eptA Gene Is Not Induced in Colistin-Resistant Spontaneous Mutants

The results described above suggest that *P. aeruginosa* is more prone to acquire mutations leading to the induction of the *arn* operon rather than the *eptA* gene, leading to postulation that *eptA* could be poorly connected to *P. aeruginosa* regulatory networks. However, random mutagenesis by transposon insertion is strongly biased toward loss-of-function mutations, as it often results in gene inactivation. While this can be useful to identify negative regulators (e.g., transcriptional repressors), it makes the identification of positive regulators much less probable. This is particularly true in our case, as the promoters under investigation (P*eptA* and P*arn*) were poorly active in the culture conditions used for the screening of transposon mutants (Figure 2, Figure 3 and Figure S3).

Thus, we decided to use a complementary approach by verifying whether P*eptA* induction occurs in spontaneous mutants that acquire resistance to colistin. To this aim, we selected colistin-resistant mutants of the reporter strains PAO1 P*eptA::lux* and PAO1 P*arn::lux*, and of the strain PAO1 *lux* as control, on agar plates containing colistin at 20 × MIC (10 μg/mL). The frequency of resistant mutants, calculated from 12 independent cultures, was overall comparable between the three strains (Table 2) and similar to values previously obtained for wild-type strains [16], indicating that the reporter constructs do not significantly affect the mutation rate nor probably the mutation patterns that can confer resistance.

**Figure 6:**
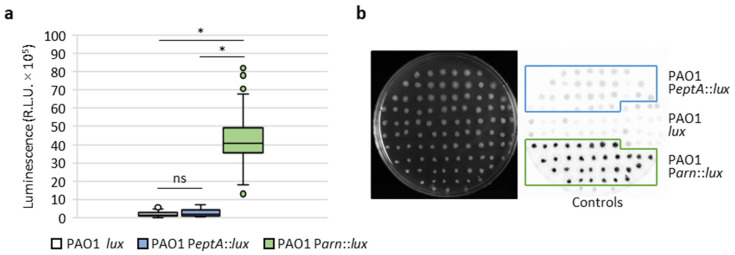
Induction of *eptA* and *arn* genes in colistin-resistant spontaneous mutants. (**a**) Box-and-whisker plots showing the luminescence emitted by 160 colistin-resistant spontaneous mutants obtained for each of the PAO1 *lux*, PAO1 P*eptA::lux*, and PAO1 P*arn::lux* strains, on LB agar plates. (**b**) Example of a screening plate, showing the growth (left image) and luminescence emission (right panel) for a subset (*n* = 32) of the colistin-resistant mutants of the above-mentioned strains. The parental (colistin-sensitive) strains PAO1 *lux*, PAO1 P*eptA::lux*, and PAO1 P*arn::lux* were included as controls. Asterisks (*) indicate a statistically significant difference (*p* < 0.001), according to the Kruskal–Wallis test. Abbreviation: ns, not significant.

For each strain, 160 colistin-resistant mutants were taken from independent replicate cultures and spotted onto LB agar plates, in order to quantify luminescence emission for each mutant. As shown in Figure 6, all the colistin-resistant mutants of the reporter strain PAO1 P*arn::lux* showed huge increases in luminescence emission, demonstrating that all these mutants had acquired mutations that (directly or indirectly) induce *arn* gene expression. In contrast, colistin-resistant mutants of PAO1 P*eptA::lux* had luminescence levels comparable to the controls, indicating that none of the mutations that conferred resistance in this group led to the induction of the *eptA* gene. Identical results were obtained when spontaneous resistant mutants were spotted on agar plates containing 10 μg/mL colistin (Figure S6), implying that *eptA* is not induced in *P. aeruginosa* even as a response to colistin exposure.

## 3. Discussion

Although the *P. aeruginosa* EptA enzyme is proficient in lipid A phosphoethanolamination, confers colistin resistance, and supports the evolution towards high-level colistin resistance when ectopically expressed from plasmids [20,21] (Figure 1), *P. aeruginosa* generally acquires colistin resistance by mutations that trigger the expression of the alternative lipid A modification system Arn, responsible for the addition of L-Ara4N to lipid A [15,16,31]. We therefore hypothesized that there could be some genetic and/or regulatory barriers to the exploitation of EptA and thus of PEtN-modified lipid A as a resistance mechanism during the natural evolution of this bacterium towards colistin resistance. To test this hypothesis, in this study we generated luminescent reporter strains to monitor *eptA* and *arn* promoter activities, and used transposon mutagenesis and a selection of spontaneous mutants resistant to colistin to compare the likelihood of mutations that induce *eptA* or *arn* gene expression and/or colistin resistance.

Transposon mutagenesis revealed that transposon insertions leading to *arn* gene induction are more probable than those activating the *eptA* promoter. Moreover, *arn*-inducing transposon insertions overall resulted in higher luminescence levels than *eptA*-inducing insertions, and increased levels of colistin resistance were observed for transposon mutants with induced *arn* gene expression but not for mutants showing P*eptA* induction (Figure 3). The genetic and phenotypic characterization of knock-out mutants showing *eptA* induction revealed that *eptA* transcription is influenced by certain metabolic stresses, such as arginine and biotin depletion (Δ*argF* and Δ*bioB*, respectively) or intracellular accumulation of the stress signal Ap4A (Δ*apaH*), and that this regulation is independent from the TCS ColRS (Figure 4, Figure 5 and Figure S5). Notably, the lack of ApaH, BioB, or ArgF had a much lower impact on *eptA* promoter activity than the exogenous signal Zn^2+^ (Figure 5), which, however, is unable to promote colistin resistance [20] (Figure S2). Since PEtN addition to lipid A in response to extracellular Zn^2+^ was confirmed by mass spectrometry [20], it is likely that the inability of Zn^2+^ to confer colistin resistance is at least partly due to the highly deleterious effect of this metal on bacterial growth under *eptA* inducing conditions (Figure S2). Interestingly, the genetic conditions identified in this study to promote *eptA* expression (*bioB*, *argF*, or *apaH* inactivation) also caused relevant growth defects (Figure 4, Figures S4 and S5), suggesting that *eptA* gene expression might mainly occur under stress conditions that do not facilitate the acquisition of an antibiotic-resistant phenotype. As an example, we can consider the case of biotin depletion (Δ*bioB* mutant), which led to the highest increase in P*eptA* activity among our knockout mutants (Figure 4 and Figure S5). Biotin can be produced by de novo synthesis or environmentally acquired; however, as biotin synthesis is expensive, bacteria generally shut down synthesis when an exogenous source of this cofactor is available [32]. Recently, biotin biosynthesis was shown to be essential during *P. aeruginosa* infection in mice in which the low biotin concentrations of human plasma were artificially mimicked [33]. This implies that mutations leading to inactivation of the biotin de novo pathway might be counter-selected in vivo, irrespective of their impact on antibiotic resistance. Another example of likely counter-selectable mutation is *apaH* inactivation, which causes accumulation of the stress signal molecule Ap4A. Additionally to impairing growth [29], Ap4A accumulation has recently been reported to increase sensitivity to killing by aminoglycosides in different bacterial pathogens, including *P. aeruginosa* [34].

Two main drawbacks of the transposon mutagenesis screening process should be noted. First, we analyzed a limited number of transposon mutants (12,000) in a strain carrying about 5500 genes [35]. According to genome-wide transposon mutagenesis projects, such a number of transposon mutants should be enough to cover about 50–60% of nonessential genes [36,37], so it is plausible that additional (direct or indirect) regulators of *eptA* may exist that were not identified in our study. Second, transposon mutagenesis generates mainly loss-of-function mutations, thus hindering the identification of genes encoding for (transcriptional) activators, especially in the case of the *eptA* promoter which is basically inactive under the culture conditions used for transposon mutant screening (Figure 2). To overcome these limitations, we also analyzed P*eptA* and P*arn* activity in hundreds of colistin-resistant spontaneous mutants selected from several independent bacterial cultures. This analysis revealed that P*arn* was induced in all colistin-resistant mutants included in our analysis, while P*eptA* was induced in none of them (Figure 6), strongly suggesting that gain-of-function mutations leading to relevant levels of *eptA* gene expression may be very rare and/or difficult to select for in *P. aeruginosa*.

In conclusion, although the mutagenesis screenings were conducted on a single strain (*P. aeruginosa* PAO1) and we cannot exclude the possibility that other strains might behave differently, our results confirm the evidence from in vitro evolution experiments and clinical isolates that lipid A aminoarabinosylation is preferred over phosphoethanolamination as a colistin-resistance mechanism in *P. aeruginosa*. Moreover, our results suggest the proposal that this could be due to the fitness costs related to the exogenous and/or endogenous signals that trigger *eptA* gene expression, and/or to the ineffectiveness of the *P. aeruginosa* regulatory networks in providing sufficient levels of *eptA* gene expression.

## 4. Materials and Methods

### 4.1. Bacterial Strains, Plasmids and Growth Media

Bacterial strains and plasmids used in this study are listed in Supplementary Materials Tables S1 and S2, respectively. Unless otherwise stated, bacteria were cultured in LB (Lennox formulation) for genetic manipulation, transposon mutagenesis, growth, and luminescence assays, or in cation-adjusted Mueller–Hinton broth CAMHB for in vitro evolution experiments and MIC assay. When required, antibiotics were added at the following concentrations for *E. coli* (the concentration used for *P. aeruginosa* is shown in brackets): ampicillin, 100 μg/mL; carbenicillin (500 μg/mL); nalidixic acid, 15 μg/mL; chloramphenicol, 30 μg/mL (375 μg/mL); tetracycline, 12.5 μg/mL (100 μg/mL); gentamicin, 10 μg/mL (50 μg/mL).

### 4.2. In Vitro Evolution Assays

To select for mutants with high-level colistin resistance, *P. aeruginosa* strains were sequentially cultured in the presence of increasing concentrations of colistin as previously described [16]. Briefly, strains were precultured in 2 mL of CAMHB at 37 °C and 250 rpm to late-exponential phase and then sequentially refreshed 1:100 in 1 mL of fresh medium in the presence of two-fold higher concentrations of colistin (from 0.25 to 64 µg/mL) as soon as cultures reached an OD_600_ > 0.5 (starting inoculum ≥ 10^7^ cells/mL). For each colistin concentration, three serial passages were performed before moving to the two-fold higher concentration. Five independent cultures were performed for each strain. Cultures showing no visible growth after five days were considered extinct [16].

### 4.3. Generation of Luminescent Reporter Strains, Deletion Mutants and Complementing Plasmids

To obtain the constructs for the generation of PAO1 P*eptA::lux* and PAO1 P*arn::lux* reporter strains, a 514-bp and a 416-bp DNA region encompassing P*eptA* or P*arn*, respectively, were PCR amplified, individually cloned into the sequencing plasmid pBluescript II KS (pBS; Table S2) and verified by DNA sequencing. Primers and restriction enzymes used for PCR and cloning are listed in Table S3. Then, P*eptA* and P*arn* were excised from pBS and directionally sub-cloned into the self-proficient integration plasmid mini-CTX1*lux* [38], yielding mini-CTX1P*eptA::lux* and mini-CTX1P*arn::lux* (Table S2). These plasmids, or the control plasmid mini-CTX1*lux*, were transferred into *P. aeruginosa* PAO1 by conjugation, and transconjugants were selected on LB agar plates containing 15 μg/mL nalidixic acid and 100 μg/mL tetracycline. The plasmid backbone was removed using the *sacB*-based suicide vector pFLP2 [39] as previously described [40], and pFLP2 was cured by plating onto LB agar plates supplemented with 10% sucrose. Carbenicillin-sensitive clones were analyzed by colony PCR to verify the insertion of the P*eptA::lux* or P*arn::lux* construct in the *P. aeruginosa* chromosome.

To obtain the constructs for the generation of *P. aeruginosa colRS, apaH, rmcA*, *bioB*, and *argF* deletion mutants (Tables S1 and S2), two DNA fragments of approximately 500 bp encompassing the upstream (↑) and downstream (↓) region of each gene of interest were PCR amplified, directionally cloned into pBS, and verified by DNA sequencing. Then, the ↑↓ fragments of each gene were excised from pBS and sub-cloned into the *sacB*-based suicide vector pDM4 [41]. Primers and restriction enzymes used for PCR and cloning are listed in Table S3. The resulting pDM4 derivatives were transferred into *P. aeruginosa* by conjugation, and transconjugants were selected on LB agar plates containing 15 μg/mL nalidixic acid and 375 μg/mL chloramphenicol. Deletion mutations were obtained by recombination and sucrose-based selection as described [40]. Gene deletions were verified by PCR and DNA sequencing.

The complementing plasmids pME*apaH*, pME*bioB*, and pME*argF* (Table S2) were generated by individually cloning the PCR-amplified coding sequence of *apaH*, *bioB*, or *argF* into the shuttle vector pME6032, under the control of an IPTG-inducible promoter [42]. All constructs were verified by DNA sequencing.

### 4.4. Growth and Luminescence Assays

For planktonic growth and promoter activity assays, reporter strains were precultured in LB and then refreshed 1:1000 in LB in the presence or absence of different ZnCl_2_ or EDTA concentrations. Bacterial cultures were incubated in 96-well microtiter plates (200 μL in each well) at 37 °C in a Spark 10M microtiter plate reader (Tecan, Männedorf, Switzerland), in order to measure growth (OD_600_) and luminescence emission (relative light units, RLU) over time. Luminescence was normalized to growth by dividing the RLU values by OD_600_ values. Area under the normalized luminescence curves was calculated using the trapezoidal method, employing GraphPad Prism software. When appropriate, growth and luminescence assays were also performed in the minimal medium M9 containing 20 mM succinate as the carbon source [43] supplemented or not with different concentrations of arginine or biotin.

For colony growth assays, reporter strains were precultured in LB, harvested by centrifugation, and resuspended in sterile saline solution at OD_600_ = 1. Serial ten-fold dilutions were prepared and 5 μL aliquots of selected dilutions were spotted onto LB agar plates containing or not 1 mM ZnCl_2_ or 1 mM EDTA. After incubation at 37 °C for 16–18 h, white light and luminescence images of plates were acquired with ChemiDoc (Bio-Rad, Segrate, Italy).

### 4.5. Transposon-Mediated Random Mutagenesis

Transposon mutagenesis was performed as previously described [44], using the pLM1 vector that holds a Tn5 transposon derivative containing an origin of replication that is functional in *E. coli*, and a gentamycin resistance gene [45]. This vector was transferred into PAO1 P*eptA::lux* and PAO1 P*arn::lux* by conjugation using *E. coli* S17.1λ*pir* as the donor strain. Transposon mutants were selected on LB agar plates supplemented with 15 μg/mL nalidixic acid and 50 μg/mL gentamycin. Colonies were picked with sterile toothpicks and spotted onto new LB agar plates. Plates were then visualized using ChemiDoc and luminescence was measured with the ImageLab software (Bio-Rad) as the adjusted volume of each clone, i.e., the sum of all intensities detected by the software within the boundaries of each colony from which the background value was subtracted. Mutants selected for further analyses were those that appeared as outliers in the box-and-whisker plots and showed a ≥2-fold increase in luminescence with respect to the median luminescence value of mutants from the same screening plate.

To map transposon insertions, genomic DNA was extracted from selected transposon mutants and digested with BamHI, XbaI, or NcoI, whose restriction sites are not present in the transposon. The digested DNA was self-ligated with T4 DNA ligase and introduced by transformation into *E. coli* S17.1 λ*pir*. Plasmid DNA was isolated from gentamicin-resistant colonies and sequenced using the Tn5 specific primers TnpRL17-1 and TnpRL13-2 (Table S3) [44].

### 4.6. MIC Assays

MIC was determined using the standard broth microdilution method. Strains were precultured in LB or CAMHB and refreshed at ca. 5 × 10^5^ cells/mL in the same medium containing increasing concentrations of colistin. MIC values were visually assessed after 24 h of growth at 37 °C under static conditions. Each strain was tested in at least three independent experiments.

### 4.7. Selection of Colistin-Resistant Spontaneous Mutants

Strains were cultured in LB broth at 37 °C until the late exponential or early stationary phase, harvested by centrifugation and resuspended in sterile saline solution at OD_600_ = 1. Serial dilutions were performed and plated onto LB agar plates to measure total numbers of CFUs. Colistin-resistant mutants were selected by plating 200 µL aliquots of the undiluted samples onto LB agar plates supplemented with 10 µg/mL colistin (20 × MIC). After 48 h of incubation at 37 °C, colistin-resistant CFUs were counted, picked with sterile toothpicks and spotted onto new LB agar plates supplemented or not with 10 µg/mL colistin. In order to evaluate the promoter activity, plates were visualized with ChemiDoc and luminescence quantified as described above (see Section 4.5). The frequency of spontaneous resistant mutants was calculated as the ratio between the number of colistin-resistant CFUs and the total number of CFUs.

### 4.8. Statistical Analysis

Statistical analysis was performed with the software GraphPad Instat, using the unpaired t test, or the Kruskal–Wallis test followed by uncorrected Dunn’s multiple comparison test for box-and-whisker plot analysis [46].

## Figures and Tables

**Table 1:** Genes disrupted in transposon mutants with increased P*eptA* activity.

Tn Mutant	Disrupted Gene (PA Number)	Insertion Site (Gene Length)	Gene Product	Function/Pathway
P34-96	*apaH* (PA0590)	95 (852 bp)	Diadenosine (Ap4A) tetraphosphatase	Ap4A degradation
P36-26	*rmcA* (PA0575)	2502 (3738 bp)	c-di-GMP phosphodiesterase	Redox regulator of c-di-GMP
P54-25	*bioB* (PA0500)	788 (1059 bp)	Biotin synthase	Biotin biosynthesis
P94-73	*argF* (PA3537)	379 (918 bp)	Anabolic ornithine carbamoyltransferase	Arginine biosynthesis
P117-04	*bioA* (PA0420)	1161 (1404 bp)	7,8-diaminononanoate (DAPA) synthase	Biotin biosynthesis

**Table 2:** Frequency of colistin-resistant spontaneous mutants.

Strain	Frequency of Resistant Mutants (±SD) ^a^
PAO1 *lux*	4.30 × 10^−8^ (±3.99 × 10^−8^)
PAO1 P*arn::lux*	6.64 × 10^−8^ (±8.32 × 10^−8^)
PAO1 P*eptA::lux*	3.72 × 10^−8^ (±8.45 × 10^−8^)

^a^ Values are the mean (±SD) of 12 biological replicates for each strain.

## Data Availability

Not applicable.

## Supplementary Materials

The following supporting information can be downloaded at: https://www.mdpi.com/article/10.3390/antibiotics12020200/s1, Figure S1: In vitro evolution assays; Figure S2: Effect of zinc on growth and colistin resistance; Figure S3: Example of a transposon mutagenesis screening plate; Figure S4: Complementation assays; Figure S5: P*eptA* induction is caused by biotin or arginine depletion; Figure S6: Induction of *eptA* and *arn* genes in colistin-resistant spontaneous mutants; Table S1: Bacterial strains used in this study; Table S2: Plasmids used in this study; Table S3: Primers used in this study. References [47,48] are cited in the supplementary materials.
