# Supplementary material for: Regulatory Landscape of the Pseudomonas aeruginosa Phosphoethanolamine Transferase Gene eptA in the Context of Colistin Resistance
Source: Antibiotics (Basel). 2023 Jan 18;12(2):200. doi: 10.3390/antibiotics12020200 (PMC9952513; doi:10.3390/antibiotics12020200)
Supplement: Supplementary file 1 [file antibiotics-12-00200-s001.zip › antibiotics-2161904-supplementary.pdf]

Regulatory landscape of the *Pseudomonas aeruginosa* phosphoethanolamine transferase gene *eptA* in the context of polymyxin resistance

Matteo Cervoni, Davide Sposato, Alessandra Lo Sciuto, Francesco Imperi

SUPPLEMENTARY MATERIAL

Table S1. Bacterial strains used in this study.

Strain	Genotype/relevant features	Source or reference
E. coli		
S17.1λ*pir*	*thi pro hsdR hsdM*$^+$ *recA* RP4-2-Tc::Mu-Km::Tn7λ*pir,* Gm^R	[47]
P. aeruginosa		
PAO1 (ATCC15692)	Reference isolate, wild type	American Type Culture Collection
PA14	Reference isolate, wild type	[48]
PAO1 Δ*arnBCA*	PAO1 derivative with an in-frame deletion of the *arnBCA* coding sequence	[16]
PA14 Δ*arnBCA*	PA14 derivative with an in-frame deletion of the *arnBCA* coding sequence	[16]
PAO1 *lux*	PAO1 derivative in which the *luxCDABE* (*lux*) operon was integrated in a neutral site of the genome	This study
PAO1 P*eptA*::*lux*	PAO1 derivative in which the *lux* operon placed under the control of the *eptA* promoter was integrated in a neutral site of the genome	This study
PAO1 P*arn*::*lux*	PAO1 derivative in which the *lux* operon under the control of the *arn* promoter was integrated in a neutral site of the genome	This study
PAO1 P*eptA*::*lux* Δ*apaH*	PAO1 P*eptA*::*lux* derivative with an in-frame deletion of the *apaH* coding sequence	This study
PAO1 P*eptA*::*lux* Δ*rmcA*	PAO1 P*eptA*::*lux* derivative with an in-frame deletion of the *rmcA* coding sequence	This study
PAO1 P*eptA*::*lux* Δ*bioB*	PAO1 P*eptA*::*lux* derivative with an in-frame deletion of the *bioB* coding sequence	This study
PAO1 P*eptA*::*lux* Δ*argF*	PAO1 P*eptA*::*lux* derivative with an in-frame deletion of the *argF* coding sequence	This study
PAO1 P*eptA*::*lux* Δ*colRS*	PAO1 P*eptA*::*lux* derivative with a deletion of the *colR* and *colS* coding sequences	This study
PAO1 P*eptA*::*lux* Δ*apaH*Δ*colRS*	PAO1 P*eptA*::*lux* Δ*apaH* derivative with a deletion of the *colR* and *colS* coding sequences	This study
PAO1 P*eptA*::*lux* Δ*bioB*Δ*colRS*	PAO1 P*eptA*::*lux* Δ*bioB* derivative with a deletion of the *colR* and *colS* coding sequences	This study
PAO1 P*eptA*::*lux* Δ*argF*Δ*colRS*	PAO1 P*eptA*::*lux* Δ*argF* derivative with a deletion of the *colR* and *colS* coding sequences	This study

Table S2. Plasmids used in this study.

Plasmid	Relevant characteristics	Source or reference
pBluescript II (pBS)	Cloning and sequencing vector; ColE1 replicon; Ap^R	Stratagene
pBSP*eptA*	pBS derivative carrying a 514-bp DNA fragment encompassing the *eptA* promoter	This study
pBSP*arn*	pBS derivative carrying a 416-bp DNA fragment encompassing the *arn* promoter	This study
pDM4	Suicide vector used for deletion mutagenesis in *P. aeruginosa*; *sacB, oriR6K*; Cm^R	[41]
pDM4Δ*apaH*	pDM4 derivative for in-frame deletion of the *apaH* coding sequence	This study
pDM4Δ*rmcA*	pDM4 derivative for in-frame deletion of the *rmcA* coding sequence	[26]
pDM4Δ*bioB*	pDM4 derivative for in-frame deletion of the *bioB* coding sequence	This study
pDM4Δ*argF*	pDM4 derivative for in-frame deletion of the *argF* coding sequence	This study
pDM4Δ*colRS*	pDM4 derivative for deletion of the *colR* and *colS* coding sequences	This study
pFLP2	Broad host range vector for site specific FLP-mediated recombination; *sacB*, Ap^R/Cb^R	[39]
mini-CTX1*lux*	Self-proficient integration vector with *tet*, Ω-FRT-*attP*-MCS, *ori*, *int*, and *oriT*, carrying the *lux* operon; Tc^R	[38]
mini-CTX1P*eptA*::*lux*	mini-CTX1*lux* derivative carrying a 514-bp DNA fragment, encompassing the *eptA* promoter, cloned upstream of the *luxCDABE* genes	This study
mini-CTX1P*arn*::*lux*	mini-CTX1*lux* derivative carrying a 416-bp DNA fragment, encompassing the *arn* promoter, cloned upstream of the *luxCDABE* genes	This study
pME6032	IPTG-inducible expression vector; $lacI^Q$, Tc^R	[42]
pME*mcr-1*	pME6032 derivative containing the *mcr-1* coding sequence downstream of the IPTG-inducible promoter	[21]
pME*eptA*	pME6032 derivative containing the *eptA* coding sequence downstream of the IPTG-inducible promoter	[21]
pME*apaH*	pME6032 derivative containing the *apaH* coding sequence downstream of the IPTG-inducible promoter	This study
pME*bioB*	pME6032 derivative containing the *bioB* coding sequence downstream of the IPTG-inducible promoter	This study
pME*argF*	pME6032 derivative containing the *argF* coding sequence downstream of the IPTG-inducible promoter	This study
pLM1	Vector containing the Tn5 transposon, used for transposon mutagenesis; Gm^R	[45]

Table S3. Primers used in this study.[a]

Primer	Sequence (5'-3')[b]	Restriction sites[c]	Application
P*eptA*_CTX*lux*_FW	ccGCTCGAGGAAATTCACAGAAG	XhoI	Generation of mini-CTX1P*eptA*::*lux*
P*eptA*_CTX*lux*_RV	cggaaTTCGACATGGGCACGGATC	EcoRI	
P*arn*_CTX*lux*_FW	ccgctcgAGTGGTCGCCGGCAGCC	XhoI	Generation of mini-CTX1P*arn*::*lux*
P*arn*_CTX*lux*_RV	cggaatTCCAGTGACATGTAATGAAGC	EcoRI	
miniCTX*lux* check	GGAAAGATTTCAACCTGGCC		Verification of reporter strains
TnpRL13-2	CAGCAACACCTTCTTCACGA		Sequencing of transposon insertion sites
TnpRL17-1	AACAAGCCAGGGATGTAACG		
apaH↑_FW	gctctaGAACAACTCGACCTGGCCG	XbaI	Generation of pDM4Δ*apaH*
apaH↑_RV	cggaatTCGCCTACCGCGTAGACC	EcoRI	
apaH↓_FW	cggaattCACGCCCGCATGAATCCC	EcoRI	
apaH↓_RV	cccctcgAGCCGATGGCCCGGAAGC	XhoI	
bioB↑_FW	gctctaGAATGTGCCCGAATGAAACC	XbaI	Generation of pDM4Δ*bioB*
bioB↑_RV	cgggatCCAGTCGTGACGGGTGGC	BamHI	
bioB↓_FW	cgggatCCCGCAGGCCGAAAAGGAC	BamHI	
bioB↓_RV	cccctCGAGGGCCAGCTCCAGTTC	XhoI	
argF↑_FW	gctctAGATGGTTCTCGTCGCTGAG	XbaI	Generation of pDM4Δ*argF*
argF↑_RV	cggaatTCCATAAACGAGAGAAAGTGC	EcoRI	
argF↓_FW	cggaatTCGAACACGCCCATTACGC	EcoRI	
argF↓_RV	ccccTCGAGCAGCAGCAACAGCG	XhoI	
colR↑_FW	gctctaGAACAGTCCGGCGATGGAG	XbaI	Generation of pDM4Δ*colRS*
colR↑_RV	cggaatTCTTCGACCACCAGTATTC	EcoRI	
colS↓_FW	cggaaTTCGAAGTTTTACTCGATGTTG	EcoRI	
colS↓_RV	cccctcgAGGATCACGCTGTCCCGC	XhoI	
*apaH*_pME6032_FW	CGGAATTCCGTACCGGGAGCGCTG	EcoRI	Generation of pME*apaH*
*apaH*_pME6032_RV	GGGGTACCTGTGTACAGGG	KpnI	
*bioB*_pME6032_FW	CGGAATTCGAAGGAATCTTCCCCATG	EcoRI	Generation of pME*bioB*
*bioB*_pME6032_RV	GGGGTACCCTCCCTTCCAGTATGG	KpnI	
*argF*_pME6032_FW	cccgagctcATGAGCGTACGGCACTTTC	SacI	Generation of pME*argF*
*argF*_pME6032_RV	AGGCGAGATCTTTCAGGTTC	BglII	
M13_FW	GTTTTCCCAGTCACGAC		DNA sequencing from pBS
M13_RV	AACAGCTATGACCATG		
pME6032_FW	GCTCTCGGGTAACATCAAG		DNA sequencing from pME6032
pME6032_RV	CGGTTCTGGCAAATATTCTG		

[a] Preparative PCRs were performed using the genomic DNA of *P. aeruginosa* PAO1 as the template and high-fidelity DNA polymerase.
[b] Lowercase letters indicate the region of the primer that does not anneal to the template.
[c] Restriction sites used for cloning are underlined in the primer sequences.

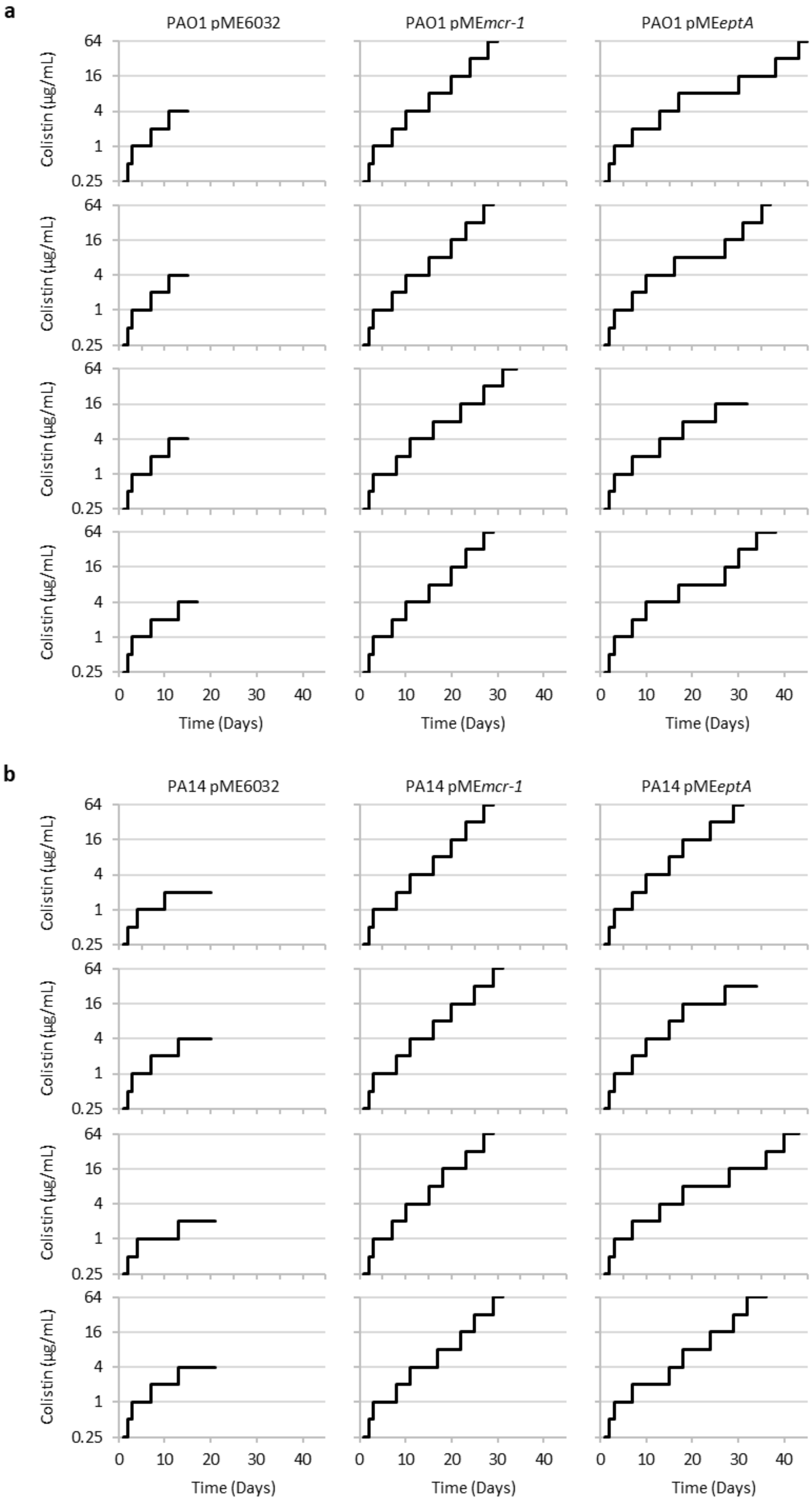

Figure S1. *In vitro* evolution assays for L-Ara4N deficient mutants of (**a**) *P. aeruginosa* PAO1 and (**b**) PA14 (Δ*arnBCA*) carrying plasmids for the ectopic expression of EptA (pME*eptA*) or MCR-1 (pME*mcr-1*), or the empty plasmid pME6032 as the control. Strains were evolved through serial passages in the presence of increasing colistin concentrations (up to 64 µg/mL). Five biological replicates (independent cultures) were analyzed for each strain; four replicates are shown in this figure, while the remaining replicate is provided as an example in Figure 1.

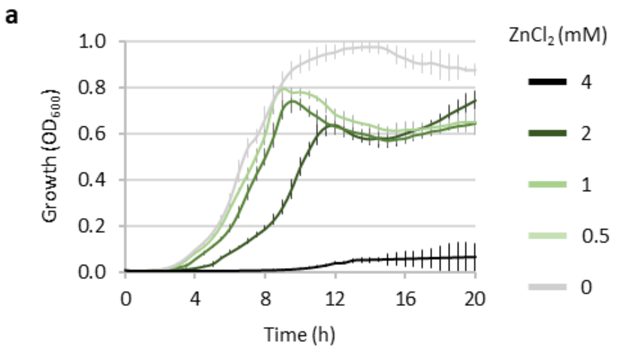

b

ZnCl$_2$ (mM)	Colistin MIC (µg/mL)	
	LB	CAMHB
0	0.5	1
0.5	0.5	1
1	0.5	1
2	0.5	1

Figure S2. Effect of zinc on growth and colistin resistance. (**a**) Growth curves of PAO1 P*eptA*::*lux* cultured in LB at 37°C in microtiter plates in the absence or in the presence of increasing $ZnCl_2$ concentrations (0-4 mM). Values are the mean (± SD) of at least three independent experiments. (**b**) Colistin MIC for PAO1 cultured in LB or in cation-adjusted MH (CAMHB) supplemented or not with $ZnCl_2$ at the indicated concentrations.

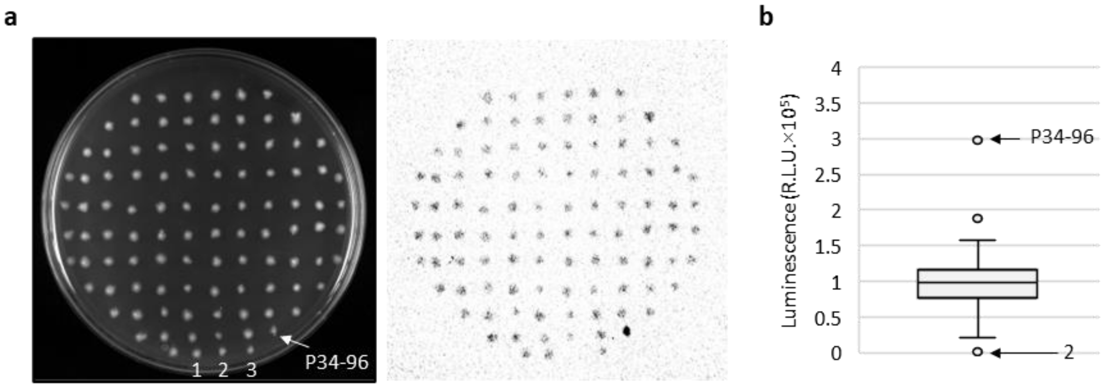

Figure S3. Example of a transposon mutagenesis screening plate. **(a)** Growth (left image) and luminescence (right image) of 97 transposon mutants of PAO1 P*eptA*::*lux* and the three controls PAO1 P*eptA*::*lux* (1), PAO1 (2), and PAO1 *lux* (3). **(b)** Box and whisker plot of the luminescence emitted by the transposon mutants and controls shown in panel a. One of the transposon mutants selected for further analyses (P34-96, see Table 1) is highlighted.

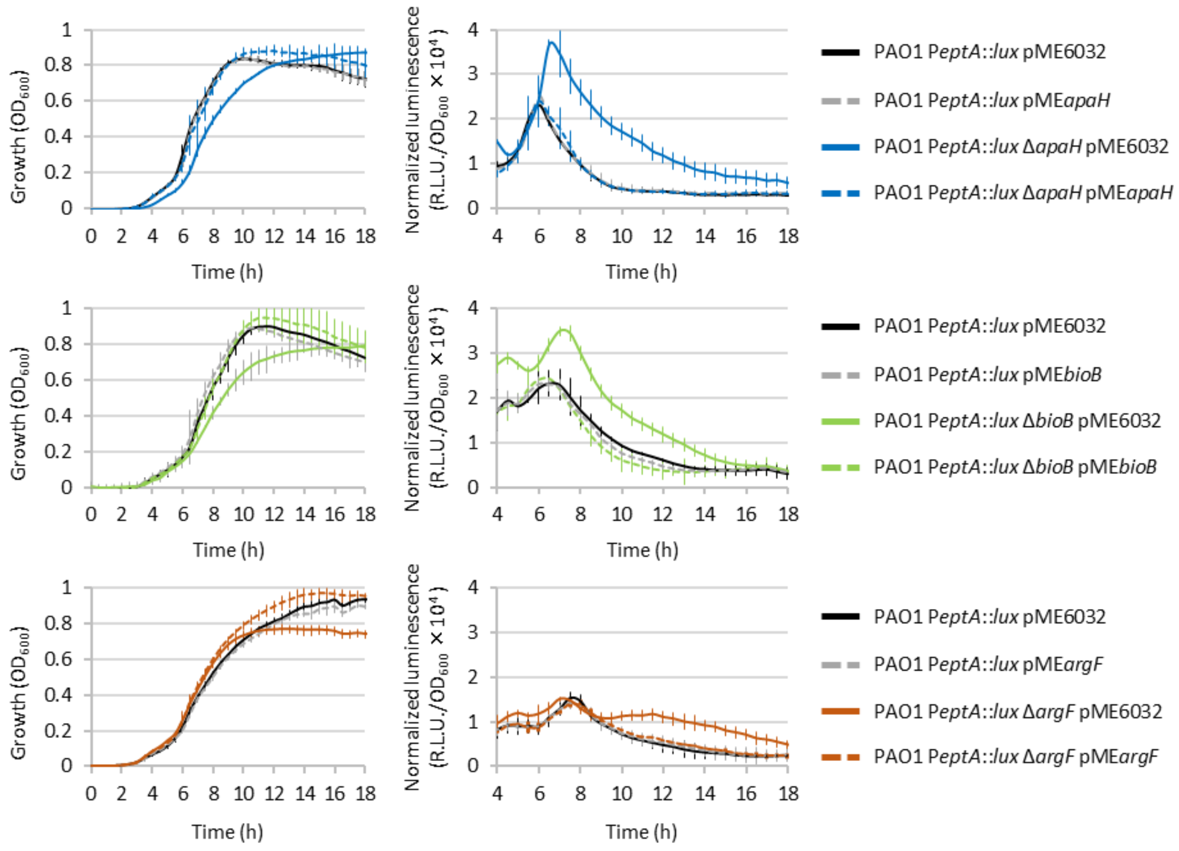

Figure S4. Complementation assays. Growth (left panels) and normalized luminescence curves (right panels) of PAO1 P*eptA*::*lux* and its derivatives deleted in *apaH*, *bioB* or *argF* harbouring the empty plasmid pME6032 or the complementing plasmids pME*apaH*, pME*bioB* or pME*argF* cultured in LB. Values are the mean (± SD) of three independent assays.

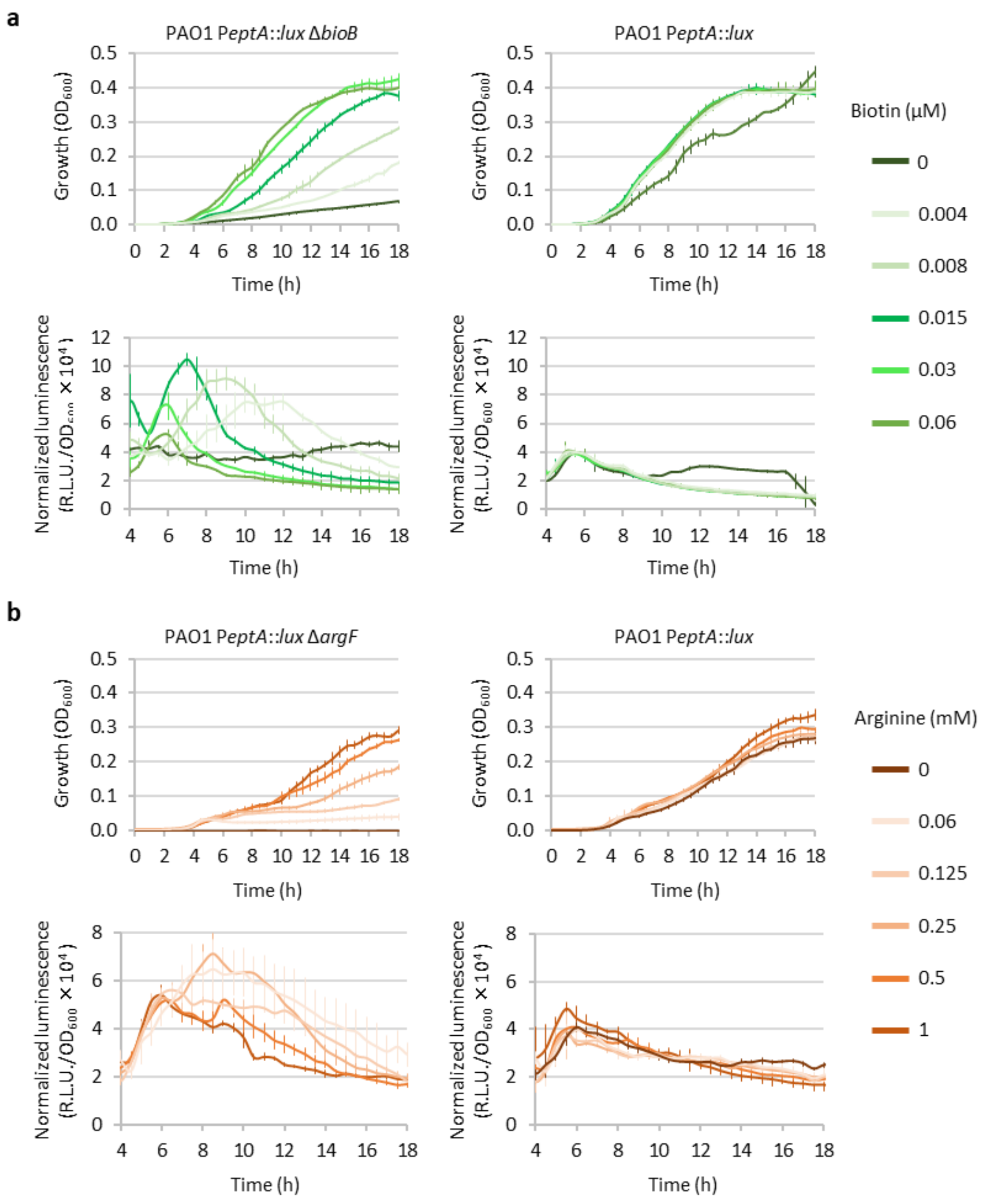

Figure S5. P*eptA* induction is caused by biotin or arginine depletion. (**a**) Growth (upper panels) and normalized luminescence curves (lower panels) of PAO1 P*eptA*::*lux* and its Δ*bioB* derivative cultured in M9 minimal medium supplemented with increasing biotin concentrations. (**b**) Growth (upper panels) and normalized luminescence curves (lower panels) of PAO1 P*eptA*::*lux* and its Δ*argF* derivative cultured in M9 minimal medium supplemented with increasing arginine concentrations. Values are the mean (± SD) of three independent assays.

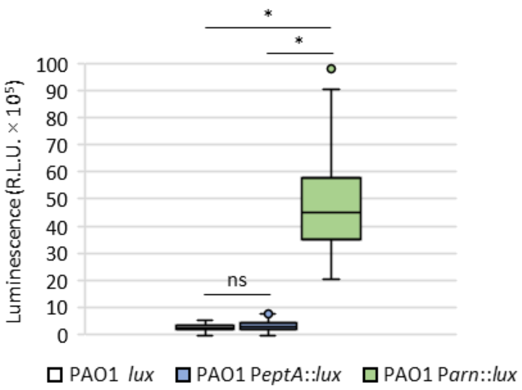

Figure S6. Induction of *eptA* and *arn* genes in colistin-resistant spontaneous mutants. Box and whisker plots showing the luminescence emitted by 160 colistin-resistant spontaneous mutants, obtained for each of the PAO1 *lux*, PAO1 P*eptA*::*lux* and PAO1 P*arn*::*lux* strains, on LB agar plates supplemented with 10 µg/mL colistin. Asterisks indicate a statistically significant difference ($P < 0.001$), according to the Kruskal-Wallis test. Abbreviation: ns, not significant.
